# Retraction Notice to: LncRNA LINC00963 Promotes Tumorigenesis and Radioresistance in Breast Cancer by Sponging miR-324-3p and Inducing ACK1 Expression

**DOI:** 10.1016/j.omtn.2025.102537

**Published:** 2025-04-11

**Authors:** Na Zhang, Xue Zeng, Chaonan Sun, Hong Guo, Tianlu Wang, Linlin Wei, Yaotian Zhang, Jiaming Zhao, Xinchi Ma

## Main text

(Molecular Therapy: Nucleic Acids *18*, 871–881; December 2019)

*Molecular Therapy Nucleic Acids* is retracting this article at the request of the editor-in-chief. A reader communicated concerns to the editorial office. These concerns are also echoed in a Pubpeer thread (https://pubpeer.com/publications/1DB54946D624578CC3AD79416F3251) in which a reader drew attention to similarities between figures in this article and an article by different authors in *Cancer Cell* (Bruno et al., 2006, Cancer Cell *10*, 473–486, https://doi.org/10.1016/j.ccr.2006.10.012). Image analysis performed by the editorial office revealed evidence of image reuse in altered fashion involving Figure 9B of this article. This reuse of data without appropriate attribution represents a severe abuse of the scientific publishing system. None of the authors responded to the notice of retraction.

